# A serotonergic perspective on depression in Parkinson’s disease: From synaptic disruption to network failure

**DOI:** 10.4103/NRR.NRR-D-25-01322

**Published:** 2026-02-05

**Authors:** Lluis Miquel-Rio, Judith Jericó-Escolar, Analia Bortolozzi

**Affiliations:** Institut d’Investigacions Biomèdiques de Barcelona (IIBB), Spanish National Research Council (CSIC), Barcelona, Spain; Institut d’Investigacions Biomèdiques August Pi i Sunyer (IDIBAPS), Barcelona, Spain; Centro de Investigación Biomédica en Red de Salud Mental (CIBERSAM), ISCIII, Madrid, Spain; Universitat de Barcelona (UB), Barcelona, Spain

For decades, the clinical identity of Parkinson’s disease (PD) has been anchored to its motor symptoms, such as tremor, rigidity, and bradykinesia, which arise from the progressive loss of dopaminergic neurons in the substantia nigra pars compacta. However, for many patients, the most debilitating aspects of the disease are psychological and emotional rather than physical. Depression, in particular, is one of the most common and impactful non-motor neuropsychiatric symptoms, affecting up to 50% of patients with PD, often emerging years before the first signs of motor impairment (Poplawska-Domaszewicz et al., 2024). The prodromal depression is not merely a reaction to a debilitating diagnosis; rather, increasing evidence suggests that it is an integral part of the underlying neurobiology of the disease. The pioneering work of Halliday et al. (1990) and, subsequently, the pathological staging model proposed by Braak et al. (2003) more than two decades ago provided the first anatomical clue, demonstrating that the accumulation of aggregated α-synuclein (α-syn) protein, the hallmark pathology of PD, begins in the lower brainstem and affects the serotonergic (5-HT) raphe nuclei long before reaching the substantia nigra pars compacta. Understanding the consequences of early disruption of the 5-HT system is essential for improving quality of life and developing therapies that treat the whole patient, not just their motor deficits.

**Figure 1:**
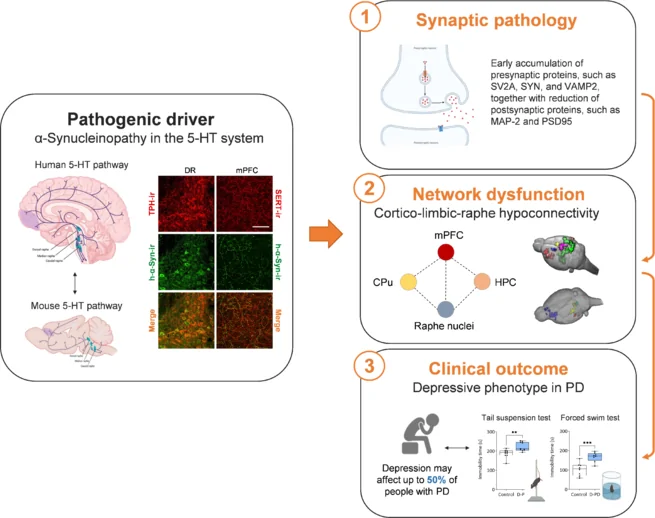
A proposed pathogenic cascade for depression in Parkinson’s disease (PD) from synaptic stress to the failure of neural circuits. The model illustrates how α-synuclein (α-syn) pathology within the serotonergic (5-HT) system acts as the primary pathogenic driver. The figure shows the initial stage, displaying confocal images that demonstrate the colocalization of human α-syn (green) with tryptophan hydroxylase (TPH)-positive 5-HT cells (red) in the dorsal raphe nucleus (DR) and serotonin transporter (SERT)-positive serotonin fibers (red) in terminal projection brain areas (medial prefrontal cortex – mPFC) of the D-PD mouse model. Scale bar: 25 μm. This initial insult triggers a downstream cascade, beginning with (1) Synaptic pathology. This stage is characterized by an early, abnormal accumulation of presynaptic vesicle-associated proteins (SV2A [synaptic vesicle glycoprotein 2A], SYP [synaptophysin], and VAMP2 [vesicle-associated membrane protein 2]), coupled with a reduction of key postsynaptic structural proteins (MAP-2 [microtubule-associated protein 2] and PSD95 [postsynaptic density protein 95]), indicating severe synaptic stress. This synaptic failure progresses to (2) Network dysfunction, where the integrity of large-scale brain circuits is compromised. This manifests as reduced functional connectivity (hypoconnectivity) within the cortico-limbic-raphe network, weakening communication between crucial hubs for emotional regulation, including the medial prefrontal cortex (mPFC), caudate-putamen (CPu), hippocampus (HPC), and the raphe nuclei. Ultimately, this circuit collapse leads to the (3) Clinical outcome: the depressive phenotype frequently observed in PD patients, often preceding motor symptoms. In the D-PD mouse model, this is evidenced by increased immobility in behavioral despair paradigms such as the tail suspension test and forced swim test. Photomicrograph component is reprinted with permission from Miquel-Rio et al. (2025). Created with BioRender.com. h-α-syn-ir: Human-α-synuclein-immunoreactivity; SERT-ir: serotonin transporter-immunoreactivity; TPH-ir: tryptophan hydroxylase-immunoreactivity.

In a recent study, Miquel-Rio et al. (2025) present a compelling preclinical model demonstrating the link between the depressive phenotype associated with oligomeric α-syn and early events at synaptic and brain network levels. By focusing on the 5-HT system, the study sheds light on a pathogenic cascade that begins with α-syn pathology and leads to the functional disconnection of brain circuits that are crucial for emotional regulation (**Figure 1**). This provides a powerful mechanistic explanation for why depression is such a fundamental aspect of PD.

The neurobiological basis of depression in PD (D-PD) is complex, but there is growing evidence pointing to the serotonergic system of the brain. The raphe nuclei, particularly the dorsal raphe nucleus (DR), which is the main source of 5-HT innervation throughout the brain, are among the first regions affected by the pathological spread of α-syn. Deficits in 5-HT signaling are notably implicated in the neuropathology of anxiety and depression. In line with this view, several neuroimaging studies have reported a positive correlation between decreased 5-HT neurotransmission and the severity of depressive and anxious symptoms in PD (Maillet et al., 2016; Politis et al., 2010). This is most likely due to the pathological changes in 5-HT neurons in the raphe nuclei. However, the precise mechanisms by which α-syn accumulation in 5-HT neurons translates into a depressive phenotype remain unclear. Two complementary studies conducted by Miquel-Rio and colleagues (2022, 2025) directly address this gap by generating a mouse model in which human α-syn is expressed in 5-HT neurons of dorsal raphe nucleus at a level comparable to that seen in PD patients with α-syn gene duplications/triplications. This specific approach allows for detailed investigation of the consequences of 5-HT-centric α-synucleinopathy, providing an effective model of the early stages of D-PD before widespread neurodegeneration and dopaminergic loss occur.

The most striking findings of the study concern the profound and progressive synaptic pathology that develops in the brain regions innervated by the affected 5-HT neurons, such as the medial prefrontal cortex (mPFC), caudate-putamen, and hippocampus. The researchers mapped a series of changes in key synaptic proteins, revealing a complex picture of early dysregulation. Notably, 8 weeks after the onset of human α-syn accumulation in 5-HT neurons with no apparent neurodegeneration, they observed a significant increase in the levels of several presynaptic vesicle-associated proteins, including synaptic vesicle glycoprotein 2A (SV2A), synaptophysin, and vesicle-associated membrane protein 2 (VAMP2). This augmentation appears to contradict the conventional narrative of neurodegeneration, which typically involves synaptic loss. Instead, these findings suggest an early pathogenic process characterized by impaired synaptic vesicle trafficking and axonal transport. The simultaneous increase in the motor protein dynein in the mPFC further supports this hypothesis, pointing to a possible “jam” of synaptic components within the axons. α-syn is known to interfere with microtubule-based transport, and this study provides evidence that this process is a critical early event in the 5-HT system, preceding eventual synaptic and axonal degeneration. Indeed, studies using aged PD mice (17–20 months old) revealed a decrease in the density of certain presynaptic markers. Furthermore, presynaptic pathology was accompanied by clear signs of postsynaptic damage. Levels of microtubule-associated protein 2, which is crucial for dendritic structure, and of postsynaptic density protein 95 (PSD95), which is a key scaffolding protein at the postsynaptic level, were both significantly reduced (**Figure 1**). Together, these findings paint a picture of a synapse under severe stress, which is struggling to maintain its structural integrity and function.

Miquel-Rio et al. (2025) confirmed these preclinical findings by analyzing post-mortem human brain tissue. They observed a comparable pattern in the dorsolateral prefrontal cortex (dlPFC) and caudate nucleus (CAU) of PD patients in the early Braak stages (2–3) of the disease, characterized by elevated levels of SV2A, synaptophysin, and vesicle-associated membrane protein 2 proteins. By contrast, they found a significant reduction in these presynaptic markers and in PSD95 level, which is consistent with the widespread synaptic loss observed in the dorsolateral prefrontal cortex and CAU of patients in the late stages (Braak 5–6) (Frigerio et al., 2024). This translational evidence is compelling as it suggests that the D-PD mouse model accurately captures an early, dynamic phase of synaptic pathology in PD that has previously been difficult to observe in humans. In this phase, synaptic dysfunction occurs prior to overt degeneration.

Although synaptic pathology provides an explanation at the cellular level, depression is ultimately a disorder of brain circuits. Another important contribution of the study was establishing a link between these synaptic changes and large-scale network dysfunction using resting-state functional magnetic resonance imaging. The results were unequivocal: α-syn accumulation in the 5-HT system led to a significant reduction in functional connectivity, or “hypoconnectivity”, across the entire cortico-limbic-raphe network. Using seed-based analyses, the researchers identified weakened functional coupling between critical nodes of emotional and cognitive control. For example, the mPFC showed reduced connectivity with the hippocampus, nucleus accumbens, and the raphe nuclei themselves. The caudate-putamen, a key hub for motor and non-motor function, displayed widespread disconnection from cortical, thalamic, and limbic partners, including the amygdala, habenula, retrosplenial cortex, globus pallidus, and substantia nigra reticulata. This functional evidence establishes a link between molecular pathology and behavioral phenotype. The observed hypoconnectivity mirrors the findings of numerous clinical neuroimaging studies in patients with D-PD, which have consistently reported altered connectivity within cortico-limbic networks (Hu et al., 2015; Qiu et al., 2021). The model proposed by Miquel-Rio et al. (2025) suggests that the progressive loss of 5-HT fiber integrity, driven by defects in axonal transport induced by the accumulation of oligomeric forms of α-syn, effectively weakens communication between these brain regions. This network collapse disrupts the ability of the brain to regulate mood and emotional responses, providing a direct neurobiological basis for the depressive symptoms that characterize PD. Importantly, a marked reduction in reciprocal connectivity between the mPFC and DR has been observed. The altered functionality of this top-down circuit, and *vice versa*, has been directly linked to the neurobiology of depression and its improvement with antidepressant treatments. Therefore, the study also mapped cellular activity by examining the expression of Egr-1, an early response gene. This revealed increased activity in corticolimbic areas, such as the mPFC and amygdala, alongside reduced function of the raphe nuclei. This could represent a maladaptive compensatory response to the failure of 5-HT input.

The implications of this work are significant, as they offer a new perspective on the prodromal phase of D-PD and highlight new avenues for diagnosis and treatment. The study posits a clear pathogenic sequence: α-syn aggregation in 5-HT neurons initiates a cascade of synaptic dysregulation, characterized by altered axonal transport and an abnormal accumulation of presynaptic proteins, which in turn leads to the functional decoupling of cortico-limbic circuits, ultimately manifesting as a depressive phenotype. This model shifts the focus from neuronal death to earlier, more subtle processes of synaptic and network dysfunction. From this perspective, early changes in synaptic proteins such as SV2A or in functional connectivity could represent valuable biomarkers. Positron emission tomography imaging of SV2A is already being explored as a clinical marker for synaptic density in neurodegenerative diseases, and the findings of this study suggest that it could be particularly useful for tracking early synaptic changes in PD. Similarly, resting-state functional magnetic resonance imaging could identify a pattern of cortico-limbic hypoconnectivity that predicts the development of depression in individuals at risk for PD. Furthermore, this mouse model provides a powerful platform for testing novel therapeutic interventions. Rather than waiting for irreversible neuronal loss, future strategies could target the earliest stages of this pathogenic cascade. Interventions aimed at clearing α-syn aggregates, restoring axonal deficits, or preserving synaptic integrity could potentially prevent or alleviate depressive symptoms. For example, therapies that improve synaptic plasticity, such as the antidepressant ketamine, an open channel blocker of ionotropic glutamate receptors N-methyl-D-aspartate, could counteract the synaptic and circuit deficiencies observed in the D-PD model. In fact, a single dose of ketamine (10 mg/kg, intraperitoneally) was effective in restoring levels of brain-derived neurotrophic factor, microtubule-associated protein 2, and PSD95, as well as improving the balance of excitatory/inhibitory responses in the mPFC. This reversed the depressive phenotype in the D-PD mouse model (Sancho-Alonso et al., personal communication). In line with this observation, several ongoing clinical trials are investigating the potential of ketamine as a treatment for depression in PD (Clinical Trials IDs NCT04944017 and NCT06231563). By focusing on preserving 5-HT system connectivity throughout the brain, we may develop disease-modifying treatments for one of the most prevalent and challenging non-motor neuropsychiatric symptoms of PD. Therefore, to fully address this pathogenic cascade, a dual therapeutic approach is likely to be required, reflecting the current diversity in the pipeline of clinical trials for PD (McFarthing et al., 2024). First, disease-modifying therapies aimed at reducing the burden of α-syn, such as monoclonal antibodies (e.g., prasinezumab) or aggregation inhibitors, are essential to halt the initial trigger of synaptic stress in 5-HT neurons. Second, subsequent symptomatic interventions must go beyond standard selective serotonin reuptake inhibitors, which often fail when 5-HT terminals are compromised. This includes 5-HT receptor modulators (e.g., 5-HT_1A_ agonists or 5-HT_2A_ inverse agonists such as pimavanserin) that can bypass presynaptic degeneration to stabilize network connectivity, along with synaptic stabilizers such as ketamine. However, it is important to recognize the limitations of this preclinical work, including its focus on male mice, given that sex differences in non-motor symptoms of PD are known to exist. Notably, our preliminary data suggest that despite comparable α-syn accumulation in 5-HT neurons, female mice exhibit a distinct behavioral phenotype characterized by anhedonia and anxiety, accompanied by contrasting alterations in synaptic protein compared to males. Future studies, including female models, will be essential for a comprehensive understanding.

In conclusion, the research by Miquel-Rio et al. (2025) provides a vital window into the earliest neurobiological events that underlie depression in PD. By elegantly connecting α-syn pathology in the 5-HT system to a cascade of synaptic failure and network collapse, their work underscores the necessity of looking beyond the dopaminergic system and motor symptoms to understand and treat the full spectrum of this devastating disorder. It charts a clear path forward, suggesting that the key to alleviating the psychological and emotional burden of PD may lie in regenerating not only lost neurons, but also lost connections.

*This work was supported by MCIU/AEI/FEDER, UE grant PID2022-141700OB-I00 (MCIN/AEI/10.13039/501100011033), AGAUR 2021-SGR-01358, Catalan Government, and CB/07/09/0034 Center for Networked Biomedical Research on Mental Health (CIBERSAM) (to AB).*

*JJE is a recipient of a predoctoral fellowship (2025 FI-AGAUR) from the Catalan Government (AGAUR, Generalitat de Catalunya).*

*Presentation at a meeting: Some of the data discussed here was presented at the Gordon Research Conference (GRC) Parkinson’s disease, Waterville Valley, NH, USA, June 8–13, 2025.*
